# Misty Watercolor Memories, Biochemically Speaking

**DOI:** 10.1371/journal.pbio.0030304

**Published:** 2005-08-23

Eyewitness testimony has a unique ability to convince juries. The attorney asks the witness to identify the guilty party. The witness points to the defendant, the crowd gasps, and the judge pounds her gavel, demanding order in the court. The jurors casually scribble something in their notes, and everybody knows that the fate of the accused has been sealed. But how reliable is a witness's memory, especially after rehearsing the testimony ad nauseam with a team of lawyers? When a witness presents testimony, is she really remembering the event, or is she remembering something she remembered? Does the initial memory remain intact, or does it degrade like a copy of a copy?

The status of witness testimony in court is just one reason neuroscientists want to understand the biochemical underpinnings of memory formation. Consolidation, the process of new memory formation that takes place in the hippocampus, requires certain proteins. Reconsolidation, the reactivation of these memories in the amygdala, requires a different set of proteins. In the past, neuroscientists hypothesized that reconsolidation might allow old and new memories to link up. A new study by Cristina Alberini and colleagues provides evidence that when rats link new memories to old, the molecular basis of this process actually resembles consolidation.

To manipulate lab rat memories, the researchers used constructions called inhibitory avoidance apparatuses. The first apparatus had two tiny rooms: a well-lit safe room and a pitch-black electric-shock room. Rats spent ten seconds in the first room, the researchers flipped on a light, and the rats entered the shock chamber. Alberini and colleagues knew that the rats had formed a new memory when they hesitated to enter the dark room.

Rats then entered a second apparatus decorated differently from the first apparatus. The safe room smelled of perfume, the walls displayed striped wallpaper, and the floor was made from smooth plastic. For rats in the second apparatus, the researchers flipped on a light but did not let the rats pass into the shock room. Alberini and colleagues deduced that the rats had compiled their memories of both the first and second apparatuses when they hesitated to enter the second dark room during a final test.

The researchers found that rats injected with anisomycin, a drug that inhibits protein synthesis, could not form a new memory of the second apparatus and sometimes forgot the first. This showed that, as predicted, both the formation of new memories and the reconsolidation of old memories require protein synthesis. The researchers demonstrated the distinction between the processes of consolidation and reconsolidation by showing that rats require a certain protein in the hippocampus only for memory consolidation and the same protein in the amygdala only for reconsolidation.

Using a combination of proteins that took advantage of the differences between consolidation and reconsolidation, the researchers inhibited either the rats' consolidation mechanism or the reconsolidation mechanism. Then, Alberini and colleagues tested the rats' ability to link their memory of the first apparatus to their exposure to the second. Upon repeated trials, the rats with blocked reconsolidation pathways successfully linked memories of both apparatuses, while the rats with blocked consolidation pathways did not. Therefore, the consolidation pathway, and not the reconsolidation pathway, plays a role in memory linkage. [Fig pbio-0030304-g001]

**Figure pbio-0030304-g001:**
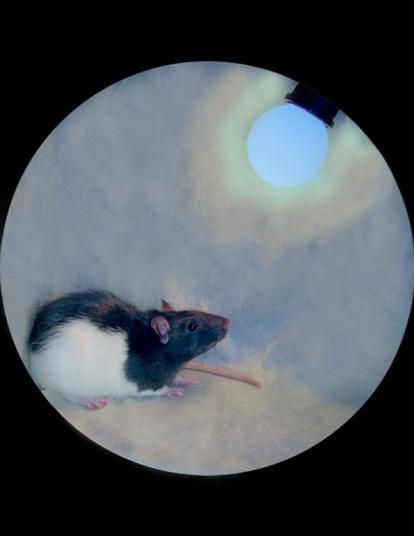
Training rats to associate light with a traumatic experience helped researchers identify the mechanisms that allow the brain to link new memories with recollections. (Sophie Tronel and Ryan Corces-Zimmerman)

As a cautionary word, the researchers emphasized that their results applied to the fear-based memories created by the electric shock. Future studies may reveal if other types of memory yield the same results.

